# Resorcinol ninhydrin complex: 1,5,9-trihy­droxy-8-oxatetra­cyclo­[7.7.0.0^2,7^.0^10,15^]hexa­deca-2,4,6,10(15),11,13-hexaen-16-one

**DOI:** 10.1107/S1600536812014249

**Published:** 2012-04-06

**Authors:** T. Uma Devi, S. Priya, G. Kalpana, S. Selvanayagam, B. Sridhar

**Affiliations:** a Department of Physics, Government Arts College for Women, Pudukkottaii 622 001, India; b Department of Physics, Cauvery College for Women, Tiruchirappalli 620 018, India; c Department of Physics, Shivani Institute of Technology, Tiruchirappalli 620 009, India; d Department of Physics, Kalasalingam University, Krishnankoil 626 126, India; e Laboratory of X-ray Crystallography, Indian Institute of Chemical Technology, Hyderabad 500 007, India

## Abstract

In the title compound, C_15_H_10_O_5_, the cyclo­penta­none (r.m.s. deviation = 0.049 Å) and oxolane (r.m.s. deviation = 0.048 Å) rings make a dihedral angle of 67.91 (4)°. An intra­molecular O—H⋯O hydrogen bond is observed. In the crystal, mol­ecules associate *via* O—H⋯O hydrogen bonds, forming a three-dimensional network.

## Related literature

For general background to ninhydrin derivatives, see: Hansen & Joullie (2005 ▶); Leane *et al.* (2004 ▶). For general background to resorcinol derivatives, see: Chen *et al.* (2011 ▶); Bao *et al.* (2010 ▶); Zheng & Wu (2007 ▶).
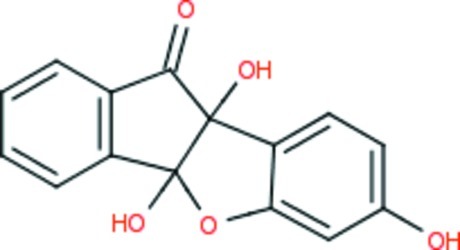


## Experimental

### 

#### Crystal data

C_15_H_10_O_5_
*M* *_r_* = 270.23
Monoclinic, 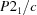

*a* = 9.1117 (5) Å
*b* = 12.2995 (7) Å
*c* = 10.1177 (5) Å
β = 91.837 (1)°
*V* = 1133.30 (11) Å^3^
*Z* = 4
Mo *K*α radiation
μ = 0.12 mm^−1^
*T* = 292 K
0.22 × 0.20 × 0.19 mm

#### Data collection

Bruker SMART APEX CCD area-detector diffractometer
12939 measured reflections
2699 independent reflections
2468 reflections with *I* > 2σ(*I*)
*R*_int_ = 0.020

#### Refinement

*R*[*F* ^2^ > 2σ(*F* ^2^)] = 0.039
*wR*(*F* ^2^) = 0.106
*S* = 1.05
2699 reflections
221 parameters
All H-atom parameters refined
Δρ_max_ = 0.31 e Å^−3^
Δρ_min_ = −0.28 e Å^−3^

### 

Data collection: *SMART* (Bruker, 2001 ▶); cell refinement: *SAINT* (Bruker, 2001 ▶); data reduction: *SAINT*; program(s) used to solve structure: *SHELXS97* (Sheldrick, 2008 ▶); program(s) used to refine structure: *SHELXL97* (Sheldrick, 2008 ▶); molecular graphics: *ORTEP-3* (Farrugia, 1997 ▶) and *PLATON* (Spek, 2009 ▶); software used to prepare material for publication: *SHELXL97* (Sheldrick, 2008 ▶) and *PLATON*.

## Supplementary Material

Crystal structure: contains datablock(s) I, global. DOI: 10.1107/S1600536812014249/bt5867sup1.cif

Structure factors: contains datablock(s) I. DOI: 10.1107/S1600536812014249/bt5867Isup2.hkl

Supplementary material file. DOI: 10.1107/S1600536812014249/bt5867Isup3.cml

Additional supplementary materials:  crystallographic information; 3D view; checkCIF report

## Figures and Tables

**Table 1:** Hydrogen-bond geometry (Å, °)

*D*—H⋯*A*	*D*—H	H⋯*A*	*D*⋯*A*	*D*—H⋯*A*
O4—H4*A*⋯O3^i^	0.90 (2)	2.02 (2)	2.877 (1)	158 (2)
O3—H3*A*⋯O4^ii^	0.89 (2)	2.18 (2)	3.013 (1)	157 (2)
O2—H2*A*⋯O1^iii^	0.87 (2)	1.92 (2)	2.746 (1)	159 (2)
O3—H3*A*⋯O2	0.89 (2)	2.33 (2)	2.667 (1)	102 (1)

Symmetry codes: (i) 
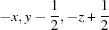
; (ii) 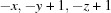; (iii) 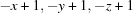.

**Fig. 1.**
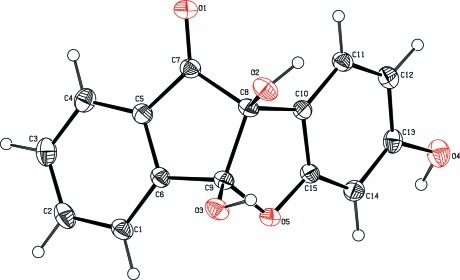
The molecular structure of the title compound, showing the atom-numbering scheme. Displacement ellipsoids are drawn at the 30% probability level

**Fig. 2.**
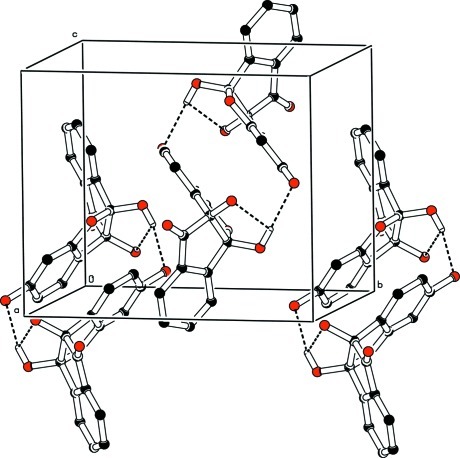
Molecular packing of the title compound, viewed along the a axis; H-bonds are shown as dashed lines forms a R22(16) dimers in unit cell. For the sake of clarity, H atoms, not involved in hydrogen bonds, have been omitted

**Fig. 3.**
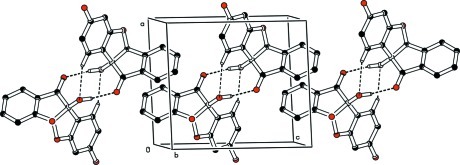
Molecular packing of the title compound, viewed along the b axis; H-bonds are shown as dashed lines forms a R22(10) dimers in unit cell. For the sake of clarity, H atoms, not involved in hydrogen bonds, have been omitted

**Fig. 4.**
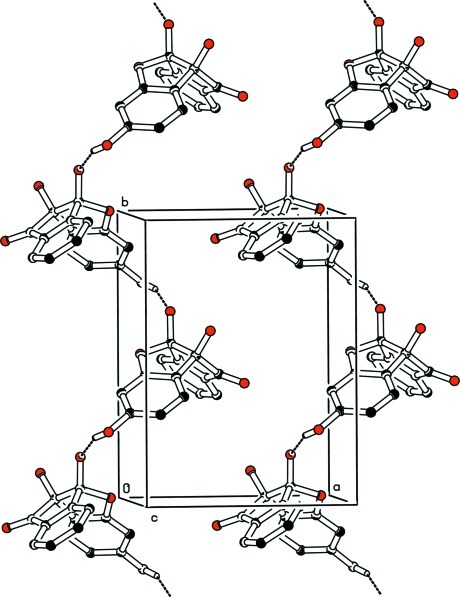
Molecular packing of the title compound, viewed along the c axis; H-bonds are shown as dashed lines forms a C(8) chain-motif in unit cell. For the sake of clarity, H atoms, not involved in hydrogen bonds, have been omitted

## supplementary crystallographic information

### Comment

Resorcinol derivatives posses cytotoxity (Zheng & Wu, 2007; Bao *et al.*, 2010) and antitumor activities (Chen *et al.*, 2011). Ninhydrin analogue was found to be an important reagent to develop finger prints on porous surface (Hansen & Joullie, 2005). Ninhydrin assay is an essential aid in the design and testing of solid dosage forms with different chitosan-drug release profiles (Leane *et al.*, 2004).In view of these importance on ninhydrin and resorcinol derivatives, we have undertaken the crystal structure determination of the present complex, and the results are presented here.

The X-ray study confirmed the molecular structure and atomic connectivity for (I), as illustrated in Fig. 1.

The plane calculation revealed that the benzene, cyclopentanone, oxolane and phenyl rings are in planar. Atoms O1, O2 and O3 deviate by 0.235 (1), 0.916 (1) and 1.166 (1) Å, respectively with respect to the fused rings of benzene and cyclopentanone. Atoms O2, O3 and O4 deviate by -1.127 (1), -0.801 (1) and 0.091 (1) Å, respectively with respect to the fused rings of oxolane and phenyl rings. The dihedral angle between the two half of the molecule with respect to C8-C9 bond is 1.5 (1)°.

The molecular structure is influenced by an intramolecular O—H···O hydrogen bonds. In the molecular packing, O—H···O hydrogen bonds involving atoms O3 and O4 link inversion-related molecules to form R_2_^2^ (16) graph-set dimer. (Fig. 2 and Table 1). In addition to this atoms, O2 and C11 form a R_2_^2^(10) graph-set motif in the unit cell with the help of intermolecular hydrogen bonds (Fig.3). A C(8) chain motif is formed in the unit cell with the help of O—H···O hydrogen bonds involving atoms O4 and O3 which results the helical shape arrangement along bc plane of the unit cell (Fig. 4).

### Experimental

A mixture of ninhydrin and resorcinol in molar ratio 1:1 were dissolved in dilute acetic medium and stirred well using a temperature controlled magnetic stirrer to yield a homogeneous mixture of solution. Then the solution was allowed to evaporate at room temperature, which yielded a crystalline adduct. Single crystals were grown by slow evaporation from ethanol.

### Refinement

All H atoms were located from a difference Fourier map and refined isotropically.

### Crystal data

**Table:**

C_15_H_10_O_5_	*F*(000) = 560
*M**_r_* = 270.23	*D*_x_ = 1.584 Mg m^−^^3^
Monoclinic, *P*2_1_/*c*	Mo *K*α radiation, λ = 0.71073 Å
Hall symbol: -P 2ybc	Cell parameters from 8468 reflections
*a* = 9.1117 (5) Å	θ = 2.2–27.1°
*b* = 12.2995 (7) Å	µ = 0.12 mm^−^^1^
*c* = 10.1177 (5) Å	*T* = 292 K
β = 91.837 (1)°	Block, colourless
*V* = 1133.30 (11) Å^3^	0.22 × 0.20 × 0.19 mm
*Z* = 4	

### Data collection

**Table:**

Bruker SMART APEX CCD area-detector diffractometer	2468 reflections with *I* > 2σ(*I*)
Radiation source: fine-focus sealed tube	*R*_int_ = 0.020
Graphite monochromator	θ_max_ = 28.0°, θ_min_ = 2.2°
ω scans	*h* = −12→11
12939 measured reflections	*k* = −16→16
2699 independent reflections	*l* = −13→13

### Refinement

**Table:**

Refinement on *F*^2^	Primary atom site location: structure-invariant direct methods
Least-squares matrix: full	Secondary atom site location: difference Fourier map
*R*[*F*^2^ > 2σ(*F*^2^)] = 0.039	Hydrogen site location: inferred from neighbouring sites
*wR*(*F*^2^) = 0.106	All H-atom parameters refined
*S* = 1.05	*w* = 1/[σ^2^(*F*_o_^2^) + (0.0594*P*)^2^ + 0.3056*P*] where *P* = (*F*_o_^2^ + 2*F*_c_^2^)/3
2699 reflections	(Δ/σ)_max_ < 0.001
221 parameters	Δρ_max_ = 0.31 e Å^−^^3^
0 restraints	Δρ_min_ = −0.28 e Å^−^^3^

### Special details

**Table:**

Geometry. All esds (except the esd in the dihedral angle between two l.s. planes) are estimated using the full covariance matrix. The cell esds are taken into account individually in the estimation of esds in distances, angles and torsion angles; correlations between esds in cell parameters are only used when they are defined by crystal symmetry. An approximate (isotropic) treatment of cell esds is used for estimating esds involving l.s. planes.
Refinement. Refinement of F^2^ against ALL reflections. The weighted R-factor wR and goodness of fit S are based on F^2^, conventional R-factors R are based on F, with F set to zero for negative F^2^. The threshold expression of F^2^ > 2sigma(F^2^) is used only for calculating R-factors(gt) etc. and is not relevant to the choice of reflections for refinement. R-factors based on F^2^ are statistically about twice as large as those based on F, and R- factors based on ALL data will be even larger.

### Fractional atomic coordinates and isotropic or equivalent isotropic displacement parameters (Å^2^)

**Table:**

	*x*	*y*	*z*	*U*_iso_*/*U*_eq_	
O1	0.55633 (10)	0.40497 (9)	0.32526 (9)	0.0416 (2)	
O2	0.38544 (11)	0.59167 (7)	0.40684 (9)	0.0382 (2)	
H2A	0.394 (2)	0.5760 (16)	0.490 (2)	0.058 (5)*	
O3	0.22262 (11)	0.65338 (7)	0.19634 (9)	0.0369 (2)	
H3A	0.219 (2)	0.689 (2)	0.272 (2)	0.080 (7)*	
O4	−0.12067 (11)	0.24783 (9)	0.55006 (10)	0.0444 (3)	
H4A	−0.175 (2)	0.2263 (19)	0.479 (2)	0.076 (6)*	
O5	0.07320 (9)	0.50826 (7)	0.24520 (8)	0.0336 (2)	
C1	0.21080 (14)	0.48007 (11)	−0.02647 (12)	0.0345 (3)	
H1	0.1252 (19)	0.5225 (14)	−0.0506 (16)	0.042 (4)*	
C2	0.27738 (16)	0.41740 (12)	−0.12105 (13)	0.0409 (3)	
H2	0.2341 (19)	0.4179 (14)	−0.2090 (18)	0.049 (4)*	
C3	0.40256 (16)	0.35623 (11)	−0.09057 (14)	0.0418 (3)	
H3	0.4472 (19)	0.3140 (14)	−0.1582 (17)	0.048 (4)*	
C4	0.46396 (14)	0.35571 (10)	0.03612 (13)	0.0366 (3)	
H4	0.5509 (18)	0.3132 (14)	0.0606 (15)	0.041 (4)*	
C5	0.39742 (12)	0.41873 (10)	0.13132 (11)	0.0301 (2)	
C6	0.27356 (12)	0.48078 (9)	0.10008 (11)	0.0285 (2)	
C7	0.44307 (12)	0.43593 (10)	0.27024 (11)	0.0302 (2)	
C8	0.32381 (12)	0.50301 (9)	0.33703 (11)	0.0283 (2)	
C9	0.22170 (12)	0.54241 (9)	0.21795 (11)	0.0289 (2)	
C10	0.22056 (12)	0.43138 (9)	0.41014 (11)	0.0278 (2)	
C11	0.24218 (13)	0.36685 (10)	0.52153 (11)	0.0310 (2)	
H11	0.3393 (17)	0.3619 (12)	0.5676 (15)	0.036 (4)*	
C12	0.12568 (14)	0.30692 (10)	0.56731 (12)	0.0336 (3)	
H12	0.1362 (17)	0.2601 (13)	0.6454 (16)	0.043 (4)*	
C13	−0.01040 (13)	0.31088 (10)	0.50070 (12)	0.0328 (3)	
C14	−0.03552 (13)	0.37657 (10)	0.39052 (12)	0.0331 (3)	
H14	−0.1314 (18)	0.3805 (13)	0.3462 (15)	0.041 (4)*	
C15	0.08212 (12)	0.43688 (10)	0.34892 (11)	0.0290 (2)	

### Atomic displacement parameters (Å^2^)

**Table:**

	*U*^11^	*U*^22^	*U*^33^	*U*^12^	*U*^13^	*U*^23^
O1	0.0309 (4)	0.0562 (6)	0.0373 (5)	0.0065 (4)	−0.0056 (4)	0.0060 (4)
O2	0.0455 (5)	0.0369 (5)	0.0315 (5)	−0.0098 (4)	−0.0097 (4)	−0.0017 (4)
O3	0.0480 (5)	0.0302 (4)	0.0321 (5)	0.0039 (4)	−0.0046 (4)	0.0019 (3)
O4	0.0441 (5)	0.0478 (6)	0.0416 (5)	−0.0145 (4)	0.0066 (4)	−0.0008 (4)
O5	0.0264 (4)	0.0455 (5)	0.0288 (4)	0.0020 (3)	−0.0033 (3)	0.0050 (3)
C1	0.0349 (6)	0.0402 (6)	0.0281 (6)	−0.0044 (5)	−0.0046 (4)	0.0013 (5)
C2	0.0495 (7)	0.0471 (7)	0.0261 (6)	−0.0107 (6)	−0.0005 (5)	−0.0029 (5)
C3	0.0504 (8)	0.0396 (7)	0.0360 (7)	−0.0074 (6)	0.0106 (6)	−0.0079 (5)
C4	0.0357 (6)	0.0338 (6)	0.0408 (7)	−0.0013 (5)	0.0063 (5)	−0.0008 (5)
C5	0.0292 (5)	0.0319 (6)	0.0293 (6)	−0.0039 (4)	−0.0003 (4)	0.0019 (4)
C6	0.0293 (5)	0.0311 (5)	0.0250 (5)	−0.0044 (4)	−0.0006 (4)	0.0009 (4)
C7	0.0269 (5)	0.0333 (5)	0.0304 (6)	−0.0031 (4)	−0.0015 (4)	0.0047 (4)
C8	0.0288 (5)	0.0309 (5)	0.0249 (5)	−0.0016 (4)	−0.0051 (4)	0.0006 (4)
C9	0.0292 (5)	0.0316 (5)	0.0258 (5)	0.0004 (4)	−0.0031 (4)	0.0009 (4)
C10	0.0277 (5)	0.0310 (5)	0.0247 (5)	0.0002 (4)	−0.0006 (4)	−0.0026 (4)
C11	0.0322 (6)	0.0333 (6)	0.0272 (5)	0.0015 (4)	−0.0031 (4)	−0.0013 (4)
C12	0.0404 (6)	0.0321 (6)	0.0282 (6)	0.0005 (5)	0.0011 (5)	−0.0002 (4)
C13	0.0354 (6)	0.0323 (6)	0.0310 (6)	−0.0049 (5)	0.0061 (4)	−0.0067 (4)
C14	0.0284 (5)	0.0397 (6)	0.0310 (6)	−0.0016 (5)	−0.0011 (4)	−0.0055 (5)
C15	0.0297 (5)	0.0334 (6)	0.0239 (5)	0.0022 (4)	−0.0013 (4)	−0.0032 (4)

### Geometric parameters (Å, º)

**Table:**

O1—C7	1.2178 (14)	C4—H4	0.975 (17)
O2—C8	1.4063 (14)	C5—C6	1.3904 (16)
O2—H2A	0.87 (2)	C5—C7	1.4683 (16)
O3—C9	1.3823 (14)	C6—C9	1.5021 (16)
O3—H3A	0.89 (2)	C7—C8	1.5379 (16)
O4—C13	1.3756 (15)	C8—C10	1.5011 (16)
O4—H4A	0.90 (2)	C8—C9	1.5749 (15)
O5—C15	1.3686 (14)	C10—C11	1.3873 (16)
O5—C9	1.4516 (14)	C10—C15	1.3890 (15)
C1—C2	1.3839 (19)	C11—C12	1.3843 (17)
C1—C6	1.3855 (16)	C11—H11	0.989 (16)
C1—H1	0.964 (17)	C12—C13	1.3930 (18)
C2—C3	1.393 (2)	C12—H12	0.979 (17)
C2—H2	0.962 (18)	C13—C14	1.3899 (18)
C3—C4	1.382 (2)	C14—C15	1.3803 (17)
C3—H3	0.960 (17)	C14—H14	0.970 (16)
C4—C5	1.3907 (17)		
			
C8—O2—H2A	109.8 (13)	O2—C8—C9	111.24 (9)
C9—O3—H3A	110.3 (15)	C10—C8—C9	101.15 (9)
C13—O4—H4A	105.4 (14)	C7—C8—C9	103.73 (9)
C15—O5—C9	107.37 (8)	O3—C9—O5	109.04 (9)
C2—C1—C6	117.74 (12)	O3—C9—C6	111.66 (9)
C2—C1—H1	119.7 (10)	O5—C9—C6	108.88 (9)
C6—C1—H1	122.5 (10)	O3—C9—C8	114.73 (9)
C1—C2—C3	121.46 (12)	O5—C9—C8	107.28 (9)
C1—C2—H2	117.4 (10)	C6—C9—C8	105.01 (9)
C3—C2—H2	121.1 (10)	C11—C10—C15	119.57 (11)
C4—C3—C2	120.81 (12)	C11—C10—C8	131.39 (10)
C4—C3—H3	119.4 (10)	C15—C10—C8	109.04 (10)
C2—C3—H3	119.8 (10)	C12—C11—C10	119.08 (11)
C3—C4—C5	117.86 (12)	C12—C11—H11	119.6 (9)
C3—C4—H4	122.6 (9)	C10—C11—H11	121.3 (9)
C5—C4—H4	119.5 (9)	C11—C12—C13	119.98 (11)
C6—C5—C4	121.15 (11)	C11—C12—H12	121.8 (9)
C6—C5—C7	109.97 (10)	C13—C12—H12	118.2 (9)
C4—C5—C7	128.83 (11)	O4—C13—C14	121.03 (11)
C1—C6—C5	120.97 (11)	O4—C13—C12	116.99 (11)
C1—C6—C9	127.29 (11)	C14—C13—C12	121.97 (11)
C5—C6—C9	111.73 (10)	C15—C14—C13	116.58 (11)
O1—C7—C5	127.04 (11)	C15—C14—H14	121.9 (9)
O1—C7—C8	124.56 (11)	C13—C14—H14	121.5 (9)
C5—C7—C8	108.39 (9)	O5—C15—C14	123.43 (10)
O2—C8—C10	117.01 (9)	O5—C15—C10	113.82 (10)
O2—C8—C7	111.12 (9)	C14—C15—C10	122.74 (11)
C10—C8—C7	111.37 (9)		
			
C6—C1—C2—C3	0.49 (19)	O2—C8—C9—O3	5.95 (14)
C1—C2—C3—C4	0.4 (2)	C10—C8—C9—O3	130.92 (10)
C2—C3—C4—C5	−0.50 (19)	C7—C8—C9—O3	−113.58 (10)
C3—C4—C5—C6	−0.34 (18)	O2—C8—C9—O5	−115.34 (10)
C3—C4—C5—C7	−177.34 (12)	C10—C8—C9—O5	9.63 (11)
C2—C1—C6—C5	−1.33 (18)	C7—C8—C9—O5	125.13 (9)
C2—C1—C6—C9	179.74 (11)	O2—C8—C9—C6	128.92 (10)
C4—C5—C6—C1	1.28 (18)	C10—C8—C9—C6	−106.11 (10)
C7—C5—C6—C1	178.80 (10)	C7—C8—C9—C6	9.39 (11)
C4—C5—C6—C9	−179.63 (10)	O2—C8—C10—C11	−62.49 (17)
C7—C5—C6—C9	−2.12 (13)	C7—C8—C10—C11	66.83 (15)
C6—C5—C7—O1	−170.36 (12)	C9—C8—C10—C11	176.52 (12)
C4—C5—C7—O1	6.9 (2)	O2—C8—C10—C15	116.84 (11)
C6—C5—C7—C8	8.49 (13)	C7—C8—C10—C15	−113.83 (10)
C4—C5—C7—C8	−174.24 (11)	C9—C8—C10—C15	−4.14 (12)
O1—C7—C8—O2	48.37 (15)	C15—C10—C11—C12	1.69 (17)
C5—C7—C8—O2	−130.52 (10)	C8—C10—C11—C12	−179.04 (11)
O1—C7—C8—C10	−84.00 (14)	C10—C11—C12—C13	0.81 (18)
C5—C7—C8—C10	97.11 (10)	C11—C12—C13—O4	178.84 (11)
O1—C7—C8—C9	167.98 (11)	C11—C12—C13—C14	−2.16 (18)
C5—C7—C8—C9	−10.91 (11)	O4—C13—C14—C15	179.85 (11)
C15—O5—C9—O3	−136.72 (9)	C12—C13—C14—C15	0.90 (18)
C15—O5—C9—C6	101.24 (10)	C9—O5—C15—C14	−170.98 (11)
C15—O5—C9—C8	−11.91 (12)	C9—O5—C15—C10	9.76 (13)
C1—C6—C9—O3	−60.94 (15)	C13—C14—C15—O5	−177.48 (10)
C5—C6—C9—O3	120.05 (11)	C13—C14—C15—C10	1.71 (17)
C1—C6—C9—O5	59.51 (15)	C11—C10—C15—O5	176.22 (10)
C5—C6—C9—O5	−119.51 (10)	C8—C10—C15—O5	−3.20 (13)
C1—C6—C9—C8	174.14 (11)	C11—C10—C15—C14	−3.04 (18)
C5—C6—C9—C8	−4.88 (12)	C8—C10—C15—C14	177.54 (10)

### Hydrogen-bond geometry (Å, º)

**Table:**

*D*—H···*A*	*D*—H	H···*A*	*D*···*A*	*D*—H···*A*
O4—H4*A*···O3^i^	0.90 (2)	2.02 (2)	2.877 (1)	158 (2)
O3—H3*A*···O4^ii^	0.89 (2)	2.18 (2)	3.013 (1)	157 (2)
O2—H2*A*···O1^iii^	0.87 (2)	1.92 (2)	2.746 (1)	159 (2)
O3—H3*A*···O2	0.89 (2)	2.33 (2)	2.667 (1)	102 (1)

Symmetry codes: (i) −*x*, *y*−1/2, −*z*+1/2; (ii) −*x*, −*y*+1, −*z*+1; (iii) −*x*+1, −*y*+1, −*z*+1.
